# Load emphasizes muscle effort minimization during selection of arm movement direction

**DOI:** 10.1186/1743-0003-9-70

**Published:** 2012-10-04

**Authors:** Wanyue Wang, Natalia Dounskaia

**Affiliations:** 1 Kinesiology Program, Arizona State University, Tempe, AZ, 85287, USA

**Keywords:** Movement cost, Interaction torque, Multi-joint, Dominant arm, Muscle energy

## Abstract

**Background:**

Directional preferences during center-out horizontal shoulder-elbow movements were previously established for both the dominant and non-dominant arm with the use of a free-stroke drawing task that required random selection of movement directions. While the preferred directions were mirror-symmetrical in both arms, they were attributed to a tendency specific for the dominant arm to simplify control of interaction torque by actively accelerating one joint and producing largely passive motion at the other joint. No conclusive evidence has been obtained in support of muscle effort minimization as a contributing factor to the directional preferences. Here, we tested whether distal load changes directional preferences, making the influence of muscle effort minimization on the selection of movement direction more apparent.

**Methods:**

The free-stroke drawing task was performed by the dominant and non-dominant arm with no load and with 0.454 kg load at the wrist. Motion of each arm was limited to rotation of the shoulder and elbow in the horizontal plane. Directional histograms of strokes produced by the fingertip were calculated to assess directional preferences in each arm and load condition. Possible causes for directional preferences were further investigated by studying optimization across directions of a number of cost functions.

**Results:**

Preferences in both arms to move in the diagonal directions were revealed. The previously suggested tendency to actively accelerate one joint and produce passive motion at the other joint was supported in both arms and load conditions. However, the load increased the tendency to produce strokes in the transverse diagonal directions (perpendicular to the forearm orientation) in both arms. Increases in required muscle effort caused by the load suggested that the higher frequency of movements in the transverse directions represented increased influence of muscle effort minimization on the selection of movement direction. This interpretation was supported by cost function optimization results.

**Conclusions:**

While without load, the contribution of muscle effort minimization was minor, and therefore, not apparent, the load revealed this contribution by enhancing it. Unlike control of interaction torque, the revealed tendency to minimize muscle effort was independent of arm dominance.

## Background

Human movements are often considered to be near-optimal, although the type of minimized movement cost remains under debate [1]. One of the most frequently considered factors is minimization of muscle effort or muscle energy [2-14]. However, a number of studies have questioned the contribution of this factor to movement formation. For instance, it was demonstrated that during point-to-point movements, the tendency to produce straight trajectory dominated the tendency to minimize muscle torque change [15], a cost function minimization of which had been proposed as the organization principle of arm movements [16]. In another study, a robotic manipulandum was used to generate a force field in which minimal metabolic energy was achieved on a curved trajectory [17]. Consistent with the finding of [15], adaptation to the force field resulted in straight movement trajectories similar to those in the null field.

Directional preferences of arm movements revealed in our group did not provide a support for minimization of muscle effort either [18,19]. In [18], subjects produced series of strokes from a circle center to the perimeter, selecting movement directions in a random order. Although instructions encouraged the uniform distribution of stroke directions, consistent directional preferences were observed. Seven cost functions were calculated for each stroke to test whether any of them can account for the observed directional preferences. Three of the cost functions addressed muscle effort for movement production in different ways. They were the change in muscle torque [16], sum of squared muscle torques across the joints [20], and inertial resistance that represented muscle effort required to generate equal hand acceleration in different directions [21,22].

The change in muscle torque and the sum of squared muscle torques did not show any anisotropic behavior. Inertial resistance was anisotropic but the minimal values were observed in directions distinct from the major preferred directions. Thus, none of the three cost functions were minimized in the major preferred directions. Rather, the directional preferences were consistent with a tendency to simplify neural control of interaction torque by actively accelerating one (leading) joint and exploiting interaction torque to produce largely passive motion at the other (subordinate) joint [23,24]. The same conclusion was obtained with the use of an unconstrained free-stroke drawing task in which strokes were not center-out but they could be initiated anywhere in the horizontal workspace in front of the subject [19].

The findings that subjects prioritize optimization of trajectory straightness and exploitation of interaction torque for movement production does not however exclude a possibility that minimization of muscle effort still influences movement formation. It is possible that there are several influential factors, and minimization of muscle effort is only one of them. Using terminology of the optimal control theory, muscle effort may be included in the resultant cost function with a certain weight. For instance, minimization of a weighted sum of angular acceleration and work produced by joint muscle torques was proposed in [14]. This composite cost function was distinguished with the use of an inverse optimal control technique that automatically inferred the weighted sum of considered optimality criteria that best fitted the experimental data.

Here, we employed another method to test the contribution of muscle effort minimization to movement formation. Namely, we examined whether load attached at the distal portion of the arm changed directional preferences revealed in [18]. Apparently, load would increase muscle effort for movement production in all directions, and therefore, the importance of muscle effort minimization would grow. In other words, load would increase the weight of muscle effort in the resultant cost function. It can be expected that muscle effort varies across movement directions. For example, inertial resistance of the arm is maximal in the two directions along the forearm longitudinal axis (*longitudinal* directions) and minimal in the two directions perpendicular to the forearm orientation (*transverse* directions) [21,25,26]. The anisotropy of inertial resistance predicts that load would change directional preferences by increasing the tendency to produce movements in the transverse directions and making movements in the longitudinal directions less attractive.

This hypothesis was tested in the present study through comparison of directional preferences during the center-out free-stroke drawing task [18] performed with and without load attached at the wrist. The task was performed with the dominant and nondominant arm to investigate whether the influence of load on the selection of movement direction depends on arm dominance. In addition to the directional biases, the dependence on distal load of a number of cost functions that may represent muscle effort was also examined.

## Methods

### Participants

Thirteen neurologically intact right-handed adults (6 males and 7 females, 23 ± 2 years of age) recruited from the university community participated in this study. Participants signed informed consent prior to participation. The institutional review board (IRB) at Arizona State University approved the experimental protocol.

### Procedure and design

The experimental procedure was similar to that described in [18,19]. Participants sat at a table the height of which was adjusted to provide arm movements approximately in the horizontal plane at the shoulder level. Movements were performed unimanually, with the dominant and nondominat arm. Slings suspended from the ceiling supported the upper arm segments to reduce muscle effort for gravity compensation. Subjects produced movements, sliding the tip of the index finger along the table surface. Movements of each arm were performed through flexion/extension of the elbow and shoulder. Motion at the trunk was prevented by restraining the torso between the table edge and the chair back. The hand was aligned with the forearm by splinting the wrist. The index finger was splinted and immobilized with respect to the hand. The finger tip was wrapped with Micropore™ paper tape to reduce friction with the table.

**Figure 1:**
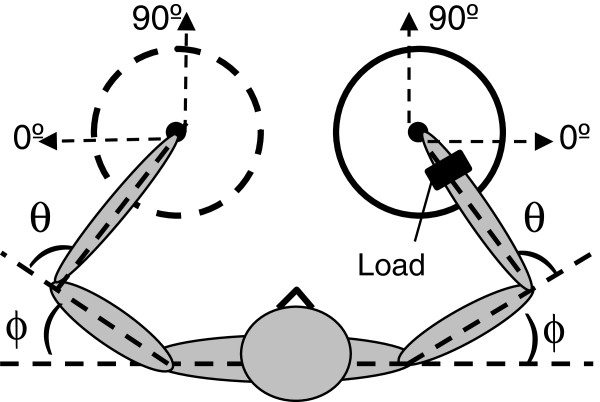
**Experimental setup and joint angles.** Subjects performed the free-stroke drawing task with either the dominant or nondominant arm within a circular workspace specified for each arm. The dashed line depicting the left circle emphasizes that the task was performed unimanually. Each arm performed the task in two load conditions, without and with load attached at the wrist. The locations of the two circle centers were defined by shoulder and elbow joint angles of ϕ = 30° and θ = 100°, respectively. Increases in values of θ and ϕ corresponded to flexion at both the elbow and shoulder joints.

A circle of a 15 cm radius and its center were depicted on the table in front of the participant. Participants produced straight fingertip strokes from the center to the perimeter of the circle. The location of the circle center was adjusted to the location of the fingertip achieved with the initial arm posture determined by the shoulder joint angle of ϕ = 30° and the elbow joint angle of θ = 100° (Figure 1). The consistency of the initial arm posture decreased differences across subjects in initial inter-segmental dynamics. The initial joint angles were chosen to allow movements from the center to the circle perimeter in all directions with minimal elastic forces that may arise at the anatomical limits of joint rotations.

A free-stroke drawing task was performed. Participants produced a series of straight center-out strokes, selecting directions of the strokes in a random order. Upon completing each stroke, participants moved the fingertip along the table surface back to the circle center to initiate a subsequent stroke. Accuracy of reaching the perimeter during center-out strokes was de-emphasized. Strokes were produced at 1.5 Hz frequency guided by a metronome. Continuous production of strokes lasted for 15 s within each trial. To encourage the uniform distribution of movement directions, participants were instructed to produce strokes in as many different directions as possible, changing directions randomly. Production of strokes in a specific order, such as around the circle clockwise or counterclockwise, was not allowed. During the experiment, participants could see their arms but fingertip motion did not leave any visible traces on the table. The purpose of the free-stroke drawing task was to reveal consistent deviations from the uniform distribution of movement directions that would be indicative of inherent directional preferences.

Performance of the task with the dominant and nondominant arm was counterbalanced across participants. Each arm performed the task in two load conditions, first with no load and then with 0.454 kg load distributed evenly around the wrist. Six fifteen-second trials of stroke production were performed by each arm in each load condition. In addition, two practice trials were performed prior to data collection in each condition. Prior to performance of the task with each arm, position of the arm while touching the circle center was recorded for 15 s. These data were later used for identification of the circle center location.

### Data collection

Arm movements were recorded with an OPTOTRAK® (Northern Digital Inc., Waterloo, Ontario, Canada) three-dimensional optoelectronic system. Seven infrared emitting diodes (IREDs) were positioned on the trunk and the two shoulders, elbows, and index fingernails. Time-varying position data were recorded at a sampling rate of 200 Hz. The collected data were filtered with a 7 Hz low-pass 4^th^-order Butterworth digital filter to remove high-frequency components that were not related to low-frequency arm motion. The data were used to determine motion of the fingertip in the horizontal plane and time-series of the shoulder (ϕ) and elbow (θ) joint angles.

### Analysis of directional preferences

The position of the finger marker on the horizontal table at each moment of time *t* was described by coordinates *x(t), y(t)* in the OPTOTRAK-determined coordinate system. Using these data, minima of fingertip marker velocity were determined and used to identify center-out strokes. Pairs of the consecutive minima were interpreted as the beginning and the end of a stroke if the first minimum occurred within a 4 cm radius from the circle center and the next minimum took place at least at 12 cm distance from the circle center. The initial and final stroke portions during which velocity was lower than 3% of its peak value were removed from the analyzed trajectories. Inclusion of sharp turns associated with reversals in movement trajectory was prevented excluding trajectory portions with curvature higher than 50 m^-1^. Curvature was computed as

(1) C=x˙⋅y˙−x˙⋅y˙/x˙2+y˙232

where $\dot{x}$,$\dot{y}$ and $\dot{x}$, *ÿ* represent the first and second derivatives of the (*x*, *y*) position data, respectively. Finally, only strokes that contained a minimum of 100 ms of data and a minimum length of 12.5 cm were considered to prevent inclusion in the analysis of shortened and incomplete strokes. 98.1% of all strokes were included in the analysis.

Length, curvature, and angular orientation of each stroke were analyzed. Since (1) provides a local characteristic of curvature at each point of the stroke, entire stroke curvature was additionally assessed as a ratio in which the denominator was the length of the straight line connecting the initial and final points of the stroke and the numerator was the maximal deviation of the stroke trajectory from this line.

The angular orientation of each stroke was determined as the direction of the line connecting the initial and final point of the stroke. The 0° orientation was assigned to movements performed in the mediolateral direction away from the body midline and 90° to movements in the anterioposterior direction away from the participant (Figure 1). The stroke orientation data obtained in the six trials by each participant in each experimental condition were used to build an individual directional histogram. Thus, four histograms were built for each participant that characterized distribution of stroke directions in the dominant and nondominant arm in the two load conditions. The histograms were produced by counting strokes within 72 bins, each of 5° width. The results were smoothed using a standard normal kernel smoothing function having a window width of 5°, yielding a probability density estimate for each orientation [27]. For visualization purposes, each individual histogram was normalized to its maximal value, resulting in values between 0 and 1.

Each individual histogram was analyzed to identify statistically significant peaks. These peaks were interpreted as directional preferences if they exceeded the mean value *H* of the histogram. The multimodal distribution within each individual histogram was analyzed with a mode existence test [28,29]. A detailed description of this analysis is presented in [18]. Briefly, this test revealed whether each peak identified in the directional histogram was either an artifact of the sample or a true feature of the population. Due to the conservative nature of tests for multimodality, peaks were considered significant at p ≤ 0.15 [28,30]. The mode existence test provided the direction and bounds of each significant peak.

As indicated in the Results section, the majority of produced strokes were fairly straight. Nevertheless, we analyzed whether directional preferences depended on the computation of stroke orientation as (1) the direction of the straight line connecting the initial and final stroke points and as (2) the direction of motion during the first 60 ms of the stroke. This analysis was performed in the same way as in our previous studies [18,19]. Like in those studies, directional preferences obtained with the two methods were approximately the same. This result is therefore not reported for the sake of conciseness.

To evaluate the dependence of directional preferences on the arm and load, *bias strength* was assessed in each directional histogram *f*(*x*) as the deviation of *f*(*x*) from its mean value *H*. Here *x* represents movement direction (0° ≤ *x* < 360°). After applying the smoothing procedure, *x* varied with an increment of 0.1° across 3601 directions. Since directional preferences were represented by histogram peaks that exceeded *H*, and values *f(x) < H* were limited by 0 (which could distort the evaluation of bias strength), only directions *x*_*i*_ (*i=1, …, N*) in which *f*(*x*_*i*_*)* >*H* were included in the computation. The bias strength was computed as:

(2) $$BS=\frac{1}{N}\frac{1}{H}\sum_{i=0}^{N}\left(f\left(x_{i}\right)-H\right)$$

where the normalization coefficients *1/N* and *1/H* were used to allow the comparison of bias strength across conditions and subjects. Computed in this way, bias strength is higher for directional histograms characterized by larger deviation of their peaks from the uniform distribution.

### Cost functions

Contribution of a factor to emergence of directional preferences was assessed with the use of a cost function representing influence of this factor on production of each stroke. Preferred directions revealed by peaks of the directional histograms were compared with the directions in which the cost function was maximized. Eight cost functions were computed. Two of them represented a tendency to exploit interaction torque by rotating either the shoulder or elbow actively and allowing predominantly passive motion generated by interaction torque at the other joint. These cost functions were included because they accounted for directional preferences in the dominant arm in our previous studies [18,19,31]. They were computed as

(3) $$I_{\mathrm{INTE}}=\frac{1}{T_{1}-T_{0}}\sum_{t=T_{0}}^{T_{1}}\frac{\left|INTE_{t}\right|}{\left|INTE_{t}\right|+\left|MUSE_{t}\right|}$$

(4) $$I_{\mathrm{INTS}}=\frac{1}{T_{1}-T_{0}}\sum_{t=T_{0}}^{T_{1}}\frac{\left|INTS_{t}\right|}{\left|INTS_{t}\right|+\left|MUSS_{t}\right|}$$

Here *T*_*0*_ and *T*_*1*_ are the time moments of the beginning and the end of the stroke*, INTE* and *INTS* are interaction torques and *MUSE* and *MUSS* are muscle torques at the elbow and shoulder, respectively, at each moment of time *T*_*0*_*≤ t ≤ T*_*1*_. *I*_*INTE*_ represented the tendency to produce predominantly passive motion at the elbow, by generating low muscle torque compared with interaction torque. Accordingly, *I*_*INTS*_ represented the tendency to produce predominantly passive motion at the shoulder. Each cost function varied between 0.0 and 1.0 with 1.0 being the optimal value representing completely passive joint motion performed with zero muscle torque. A movement was considered optimized if the value of the corresponding cost function was greater than 0.6. This threshold distinguished strokes during which motion at the corresponding joint was predominantly passive, i.e., it was generated through a larger contribution of interaction torque compared with muscle torque.

Interaction torque and muscle torque at the shoulder and elbow were computed with the use of inverse-dynamics equations of two-joint horizontal arm motion. The equations are presented in [32] as torque definition II. These equations allow computation of interaction torque, muscle torque, and net torque at each joint from angular velocity and acceleration time series obtained by differentiation of shoulder (ϕ) and elbow (θ) joint angles. Interaction torque represents the passive rotational effect attributed to reaction forces at the joints due to motion of the adjacent limb segments. Muscle torque represents the effect of muscle forces on joint rotation. Net torque is the sum effect of both interaction and muscle torque. The influence of gravitation was not considered because arm movements were performed in the horizontal plane. Anthropometric measurements including limb segment inertia, mass, and center of mass exploited for torque calculations were estimated from regression equations using the height and weight of each subject [33].

In addition to *I*_*INTE*_ and *I*_*INTS*_, six other cost functions were tested. *I*_*IR*_ assessed inertial resistance [21], *I*_*JRK*_ assessed jerk [34], *I*_*MUSC*_ assessed muscle torque change [16], *I*_*SMUS*_ assessed squared muscle torque [20], *I*_*E*_ assessed mechanical energy represented by work produced by MUS [12], and *I*_*ACC*_ assessed angular acceleration with constraints [35]. *I*_*IR*_, *I*_*JRK*_, *I*_*MUSC*_, and *I*_*SMUS*_ were tested in our previous studies [18,19]. No support for the influence of *I*_*JRK*_, *I*_*MUSC*_, and *I*_*SMUS*_ on observed directional biases and even no modulation of these costs across movement directions were found. Similar results were obtained for these three cost functions in the present study. For this reason, we chose not to present these results and to exclude these cost functions from further consideration.

Although no strong support for explaining directional biases with *I*_*IR*_ was obtained in our previous studies, this cost function was analyzed because load increased inertial resistance, and thus, it could increase the influence of this cost function on directional preferences. Specifically, load could strengthen preferences for the transverse directions in which *I*_*IR*_ was minimal and weaken preferences for the longitudinal directions in which *I*_*IR*_ was maximal [21,22,25]. Following [18], *I*_*IR*_ was defined as

(5) $$I_{\mathrm{IR}}=1.0-\frac{\left|\beta_{s}-E_{\min,IR}\right|}{\pi /2}$$

where *β*_*s*_ is the angular orientation of the stroke, and *E*_*min,IR*_ is the orientation of the minor eigenvector of the matrix *T*_*IR*_ = (*J* ')^− 1^*MJ*^− 1^. Here *J* is the 2 × 2 Jacobian matrix and *M* is the 2 × 2 matrix of the limb’s inertial properties. *J* and *M* were computed in the same form as in previous studies with the use of the initial joint angles and individual anthropometric characteristics [36,37]. The minor eigenvector of *T*_*IR*_ denotes the directions of the least inertial resistance (the two transverse directions). In these directions, *I*_*IR*_ achieves its maximal value of 1.0. Values of *I*_*IR*_ > 0.875 were used to identify strokes during which *I*_*IR*_ was optimized. This threshold determined the transverse directions ± 11.25° as the intervals of the directions optimized in terms of *I*_*IR*_.

*I*_*E*_ and *I*_*ACC*_ were considered because of the recent support for optimization of a weighted sum of these cost functions during arm movements [14]. Following that study, these two cost functions were computed as

(6) $$I_{E}=1.0-\frac{E_{s}}{\max_{s}\left(E_{s}\right)}$$

where

(7) $$E_{s}=\sum_{t=T_{0}}^{T_{1}}\left(\left|{\dot{\phi }}_{t}\cdot MUSS_{t}\right|+\left|{\dot{\theta }}_{t}\cdot MUSE_{t}\right|\right)$$

and

(8) $$I_{\mathrm{ACC}}=1.0-\frac{ACC_{s}}{\max_{s}\left(ACC_{s}\right)}$$

where

(9) $$ACC_{s}=\sum_{t=T_{0}}^{T_{1}}\left({\dot{\phi }}_{t}^{2},+,{\dot{\theta }}_{t}^{2}\right)$$

Like the other tested cost functions, *I*_*E*_ and *I*_*ACC*_ varied between 0.0 and 1.0 with 1.0 representing the optimal value. Similar to *I*_*IR*_, the threshold for them was set at 0.875.

Directional histograms of strokes optimized according to each cost function were computed as the percent of optimized strokes out of the total number of strokes in each direction. Contribution of a cost function to directional preferences was suggested if a peak of the corresponding histogram of optimized strokes coincided with one of the preferred directions represented by a peak of the directional histogram of all strokes.

### Statistical analyses

Statistical analyses were used to study the dependence of directional preferences and factors contributing to them on the arm dominance and load condition. A 2 × 2 (arm × load condition) ANOVA with repeated measures on both variables was applied to data obtained from the dominant and nondominant arm in the two load conditions, no load and with load. Characteristics analyzed were the number of produced strokes, rate of stroke production, stroke length and curvature, bias strength, mean value of each cost function computed across all strokes, and the percentage of strokes optimized in terms of each cost function. The level of significance was set at 0.05.

## Results

### Stroke characteristics

Table 1 provides mean and SD of the number of produced strokes, rate of stroke production, stroke length and stroke curvature for each arm in the no-load and with-load conditions. The number of strokes and the rate of stroke production were slightly but significantly higher in the dominant arm compared with the nondominant arm [F(1, 12) = 14.86, P < 0.01 and F(1, 12) = 14.86, P < 0.01, respectively]. Load significantly increased stroke length [F(1,12) = 5.13, P < 0.05]. Strokes performed with the nondominant arm were more curved compared with strokes performed with the dominant arm [F(1, 12) = 21.62, P < 0.01]. This result was consistent with previous findings that point-to-point movements performed by the nondominant arm were more curved than those of the dominant arm [38,39]. Other main effects and interactions for the stroke characteristics were not significant (P > 0.01), except for the load effect on stroke curvature that was marginally significant (P = 0.08).

**Table 1:** Mean and (SD) of stroke characteristics

	**Dominant arm**		**Nondominant arm**	
	**No load**	**With load**	**No load**	**With load**
Stroke number*	21.2 (2.1)	21.1 (2.3)	19.8 (2.4)	19.7 (1.9)
*Stroke rate (s*^*-1*^)*	1.4 (0.14)	1.4 (0.16)	1.3 (0.16)	1.3 (0.13)
Stroke length (cm) **	15.2 (0.80)	15.4 (0.83)	15.3 (0.80)	15.5 (0.87)
Stroke curvature*	0.040 (0.008)	0.039 (0.005)	0.049 (0.01)	0.045 (0.007)

* Significant arm effect (P<0.05); ** Significant load effect (P<0.01).

### Directional preferences

**Figure 2:**
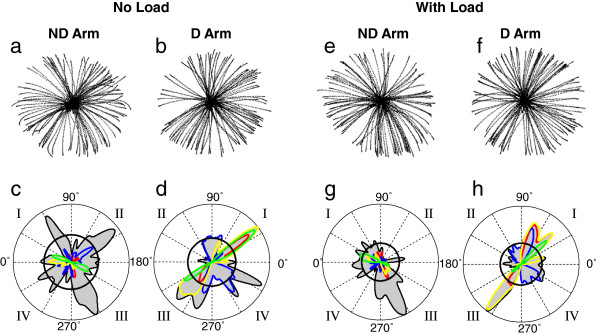
**An example of strokes and polar representations of corresponding directional histograms obtained from a representative subject during production of the task with the dominant (D) and nondominant (ND) arm without and with load.** The histograms represented by the contours of the grey areas demonstrate the frequency of stroke production in each direction. Peaks of these histograms represent preferred directions. Quadrants I, II, III, and IV of movement directions are denoted mirror-symmetrically for the D and ND arms. The histograms are overlapped with traces representing the directional histograms of strokes optimized according to *I*_*INTE*_ (blue), *I*_*INTS*_ (red), *I*_*IR*_ (green), and *I*_*E*_ (yellow). Here and in the other histograms, 0° denotes the lateral direction away from the body midline and 90° denotes the anterior direction away from the body

A representative example of stroke production by a single subject with each arm without and with load is given in Figure 2. The top panels show strokes produced in all six trials performed in each load condition by the dominant and nondominant arm. The majority of the strokes were fairly straight, which was typical for all subjects. Also, it is apparent that the distribution of the strokes across directions was not uniform in all four conditions. The corresponding histograms of stroke directions shown at the bottom (Figure 2c, d, g, h) confirm this observation. Pronounced peaks of each histogram reveal directional preferences consistent with those documented in our previous studies. In particular, each histogram contains peaks oriented in the two diagonal directions, which is similar to the results obtained in previous studies of directional preferences [18,31]. The diagonal peaks of the histograms show that the preferences in both arms were to produce movements approximately in the transverse directions (quadrants I and III) and in the longitudinal directions (quadrants II and IV). Apparently, these directions were symmetrical in the dominant and nondominant arm with respect to the anterior-posterior midline. The color traces in Figure 2 present results for cost function optimization reported further.

**Figure 3:**
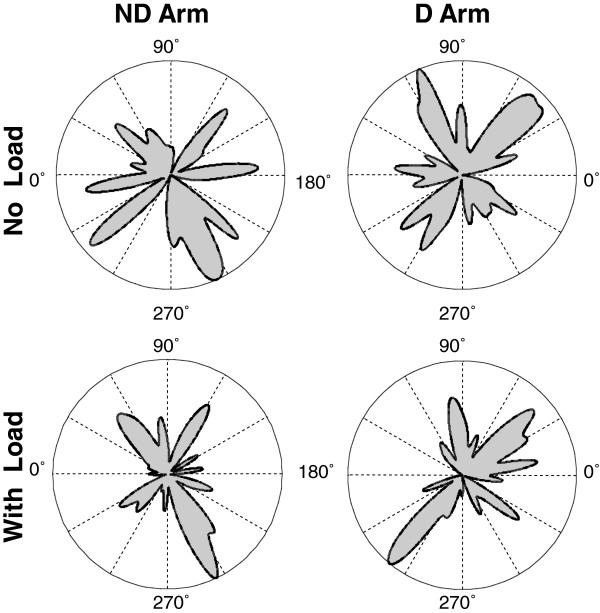
**Directional histograms of statistically significant peaks detected in all individual directional histograms of strokes produced with the dominant (D) and nondominant (ND) arm without load and with load.** The major peaks of each histogram were oriented in the diagonal directions, demonstrating that statistically significant peaks of individual histograms were also oriented mainly in these directions.

**Figure 4:**
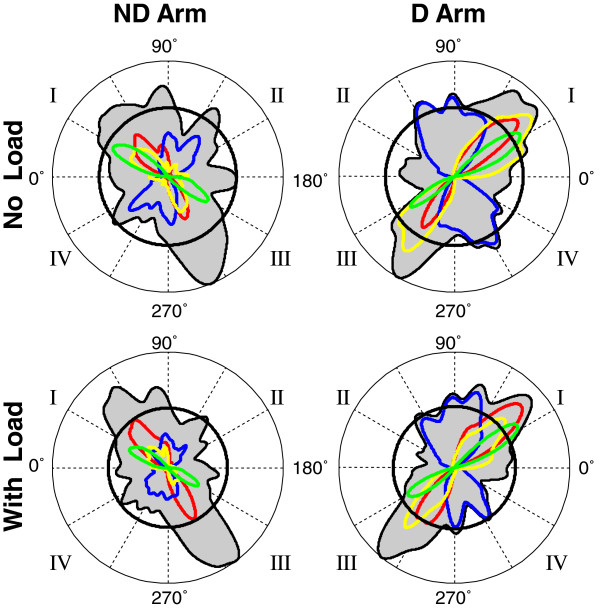
**Group histograms of stroke directions obtained with the dominant (D) and nondominant (ND) arm without and with load.** The color contours show group histograms of strokes optimized in terms of *I*_*INTE*_ (blue), *I*_*INTS*_ (red), *I*_*IR*_ (green), and *I*_*E*_ (yellow).

The preference of both arms to move in the diagonal directions was common across subjects, as was demonstrated through the analysis of statistically significant peaks. Each individual histogram had 4.2 (SD = 0.9) statistically significant peaks, and this number was not influenced by either arm dominance or the load (P > 0.1). Figure 3 presents directional histograms of the statistically significant peaks detected across all subjects in each arm and load condition. The diagonal orientations of the major peaks of the four histograms confirm that the majority of the individual histogram peaks were diagonal. The preferences to produce strokes in the diagonal directions are also apparent in group histograms built for directions of strokes produced by all subjects in each condition (Figure 4).

**Figure 5:**
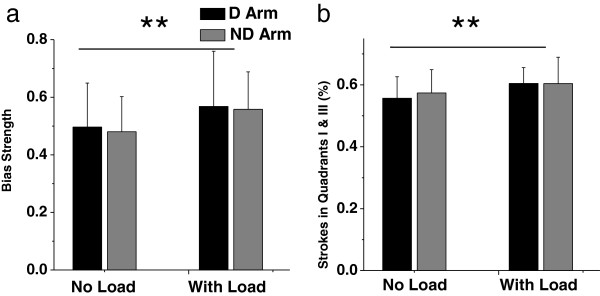
**Effect of arm and load on bias strength (a) and on the percentage of strokes produced total in quadrants I and III (b).** The load significantly increased bias strength equally in both arms. The significant main effect of load is indicated by **. The vertical bars on the top of the columns represent SD.

Visual comparison between the top and bottom group histograms in Figure 4 shows that load caused stretching of the histograms along the transverse diagonal. This load effect was supported by analysis of two characteristics, bias strength (Figure 5a) and the percentage of strokes produced in quadrants I and III out of the total number of strokes (Figure 5b). Both characteristics increased with load [F(1, 12) = 7.47, P < 0.05 and F(1,12) = 12.65, P < 0.01, respectively]. These load effects were similar for both arms. Indeed, the arm effect and interaction were not significant for both characteristics (P > 0.1). These results demonstrate that the load caused increases in the preference to produce strokes in quadrants I and III, i.e. approximately in the transverse directions.

### Cost function optimization

Here, results for optimization of five cost functions, *I*_*INTE*_, *I*_*INTS*_, *I*_*IR*_, *I*_*E*_ and *I*_*ACC*_, are reported. The color traces in Figure 2 and 4 represent directional histograms of strokes optimized according to four out of the five cost functions, excluding *I*_*ACC*_. Optimization of *I*_*ACC*_ is addressed further. Each color histogram shows the percent of strokes performed in each direction during which the corresponding cost function was nearly optimal (exceeded its threshold value). In both figures, peaks of the histograms of strokes optimized with each cost function were usually aligned with some peaks of the directional histograms, which is especially apparent for the dominant arm. Peaks of the histograms of strokes optimized with *I*_*INTE*_ were aligned with the peaks of the preferred directions along the longitudinal diagonal, i.e. the diagonal of quadrants II and IV. Peaks of the histograms of strokes optimized with *I*_*INTS*_, *I*_*IR*_, and *I*_*E*_ were aligned with the peaks of the directional histograms oriented along the transverse diagonal, i.e. the diagonal of quadrants I and III. However, it is observed in Figure 4 that the directions in which *I*_*IR*_ was optimized (the green peaks) were slightly but consistently distinct from the directions of the transverse peaks of the directional histograms. Namely, the *I*_*IR*_ optimal directions were slightly rotated clockwise in the dominant arm and counterclockwise in the nondominant arm relative to the transverse peaks of the directional histograms.

**Figure 6:**
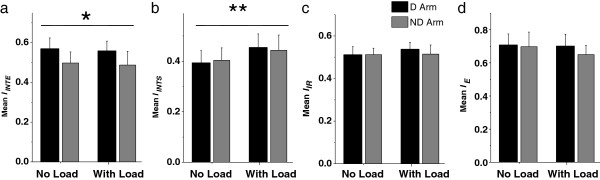
**Mean values of *I***_***INTE***_**, *I***_***INTS***_**, *I***_***IR***_**, and *I***_***E***_**during performance of the task with the dominant (D) and nondominant (ND) arm without and with load.** There was a significant effect of arm (indicated by *) on mean *I*_*INTE*_ and significant effect of load (indicated by **) on mean *I*_*INTS*_. The vertical bars on the top of the columns represent SD.

Load did not produce a significant effect on optimization of *I*_*INTE*_. The main effect of load was not significant for the mean value of this cost function computed across all directions for each subject in each condition (Figure 6a, P > 0.1). The arm effect was significant with the mean *I*_*INTE*_ being larger in the dominant arm compared with the nondominant arm [F(1,12) = 9.17, P<0.01]. The interaction was not significant (P > 0.1).

Since *I*_*INTS*_, *I*_*IR*_, and *I*_*E*_ were optimized approximately along the transverse directions, the load-related increases in the relative number of strokes produced in quadrants I and III may suggest that the mean values of *I*_*INTS*_, *I*_*IR*_, and *I*_*E*_ also increased with the load. However, this expectation was confirmed for *I*_*INTS*_ only (Figure 6b-d). Mean *I*_*INTS*_ significantly increased with load in both arms [F(1,12) = 51.31, P < 0.01], while the main effect of arm and the interaction were not significant (P > 0.1). The two main effects and the interaction were not significant for both mean *I*_*IR*_ and mean *I*_*E*_, although the main effect of load approached significance for *I*_*IR*_ [F(1,12) = 4.65, P = 0.052]. Figure 6c shows that this load effect was not consistent for both arms, causing slight increases in mean *I*_*IR*_ predominantly in the dominant arm.

The results for the mean values of the cost functions were supported by results for the percentage of strokes optimized by each cost function (Table 2). Again, only the arm effect was significant for the percentage of strokes optimized in terms of *I*_*INTE*_ that was lower in the nondominant arm, compared with the dominant arm [F(1, 12) = 14.97, P<0.01]. The load effect and the interaction were not significant (P > 0.1). The percentage of strokes optimized in terms of *I*_*INTS*_ increased with load [F(1,12) = 11.58, P < 0.01], while the arm effect and the interaction were not significant (P > 0.1). Both main effects and the interaction were not significant for the percentage of strokes optimized in terms of *I*_*IR*_ (P > 0.1). The percentage of strokes optimized in terms of *I*_*E*_ was significantly lower in the nondominant arm compared with the dominant arm [F(1, 12) = 8.35, P<0.05], while the main effect of load and the interaction were not significant (P > 0.1). Noteworthy, this characteristic had high inter-subject variability, as follows from the high SD in all four conditions (Table 2).

**Table 2:** Mean and (SD) of the portion of strokes (%) optimized with respect to each cost function

**Cost Function**	**Dominant arm**		**Nondominant arm**	
	**No load**	**With load**	**No load**	**With load**
*I*_*INTE*_ *	49.6 (9.1)	47.3 (9.0)	32.5 (12.3)	29.0 (14.4)
*I*_*INTS*_ **	19.0 (5.7)	27.0 (8.9)	18.7 (7.1)	22.9 (11.1)
*I*_*IR*_	12.7 (4.0)	13.8 (4.9)	12.7 (3.6)	10.7 (5.3)
*I*_*E*_ *	36.0 (10.8)	25.7 (23.0)	31.1 (18.1)	15.6 (9.9)

* Significant arm effect (P < 0.05); ** Significant load effect (P < 0.01).

**Figure 7:**
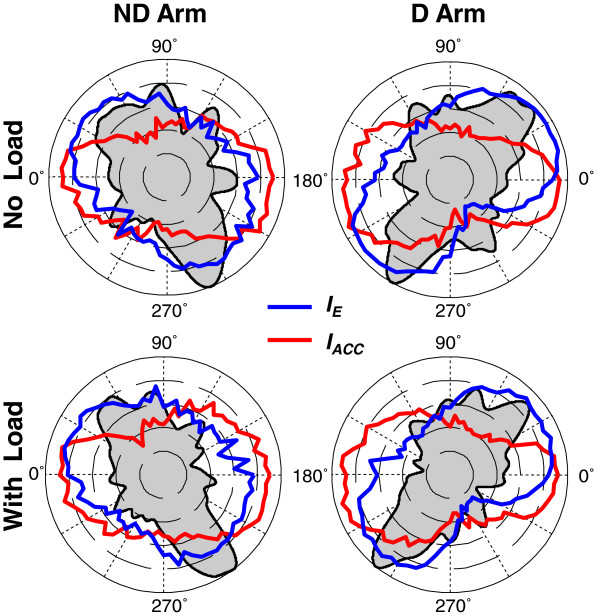
**The group directional histograms (black) overlapped with curves representing mean values of *I***_***E***_**(blue) and mean values of *I***_***ACC***_**(red)** .

The above analyses were not applied to *I*_*ACC*_ because peaks of the histograms of strokes optimized by this cost function were not aligned with the preferred directions, as observed in Figure 7. The directions in which mean *I*_*ACC*_ increased (the red contours) were not close to any major peaks of the directional histograms. Thus, the optimization of *I*_*ACC*_ did not account for any of the directional preferences. In addition, Figure 7 shows that the association of any directional preferences with the optimization of a weighted sum of *I*_*E*_ and *I*_*ACC*_[14] also has to be rejected. This figure shows that the highest values of *I*_*E*_ (the blue contours) overlapped the transverse peaks of the directional histogram, and therefore, this cost function alone could be a contributor to this directional preference. However, any weighted sum of it with *I*_*ACC*_ would have lower explanatory power. Indeed, maximal values of a weighted sum of *I*_*E*_ and *I*_*ACC*_ would be in between the directions of maximal *I*_*E*_ values and of maximal *I*_*ACC*_ values. Since the highest values of *I*_*ACC*_ were achieved in approximately lateral directions, the maximal values of the composite cost function would be rotated away from the transverse histogram peaks, and thus, would provide lower fit to the histogram peaks compared with maximal values of *I*_*E*_ alone.

## Discussion

### Effect of load on directional preferences

In our previous studies of directional preferences revealed during the free-stroke drawing task, it was found that movements in the preferred directions were performed by rotating either the shoulder or the elbow actively and by moving the other joint largely passively, by interaction torque [18,19,31,40]. This finding was predicted by the leading joint hypothesis (LJH) that suggests that during shoulder-elbow movements, muscle torque at one (leading) joint serves to generate motion and muscle torque at the other (subordinate) joint serves to control the effect of interaction torque caused by the leading joint motion [23,24]. Since control of interaction torque involves complex feedforward and feedback neural processes [41-44], the LJH predicts a tendency to minimize interaction torque control, i.e. to move the subordinate joint predominantly passively. The association of the directional preferences with the tendency to minimize active control of interaction torque can be interpreted as a tendency to minimize “neural effort” for control of inter-segmental dynamics. This interpretation was supported by marked strengthening of directional preferences caused by cognitive load [31,40].

Alternative interpretations of the directional biases were examined in the previous studies by testing whether various cost functions were optimized in the preferred directions. In particular, a number of cost functions that may represent minimization of muscle effort were tested. No evidence in support for this tendency was obtained. Here, we re-examined the contribution of the muscle effort minimization to the selection of movement direction by studying the effect of distal load on directional preferences. Apparently, distal load increases muscle effort for stroke production. It could therefore be expected that if muscle effort minimization influences selection of movement direction, load would make this influence stronger, causing more frequent selection of the directions in which muscle effort is minimal.

The results demonstrated that the load attached at the wrist caused stretching of the individual directional histograms approximately along the transverse directions, as observed in the group histograms (Figure 4). This visual observation was statistically supported by the load-related increases in bias strength and in the percentage of strokes produced in quadrants I and III (Figure 5). These results suggest that muscle effort for stroke production is minimal in the transverse directions, and the load increased the weight of muscle effort in the composite cost function, causing more frequent production of strokes in the directions of minimal muscle effort.

It can be argued that the directional biases changed because the load increased interaction torque, and thus, it enhanced the tendency to produce passive motion at the subordinate joint. However, it is unclear why the load strengthened the preference for the transverse directions in which the shoulder moved passively and made less preferred the longitudinal directions in which the elbow moved passively. Also, if the load-related changes in the directional preferences were associated with interaction torque control, differences between the dominant and nondominant arm would be observed because of the reduced proficiency of the nondominant arm in interaction torque control [38,39,45]. Such inter-arm differences were not found. First, bias strength and the percentage of strokes produced in quadrants I and III increased with load equally in both arms. Second, no significant arm effect or arm by load interaction were found for optimization of *I*_*INTS*_ that represented the tendency to actively rotate the elbow and passively rotate the shoulder.

The provided considerations show that it is unlikely that the frequency of strokes in the transverse directions increased because the load strengthened the preference to passively rotate the shoulder. Rather, the increased preference to move in the transverse directions caused by the load was associated with the increased preference to minimize muscle effort for stroke production. This preference may be limited without load, with other factors being more influential. One such factor may be the tendency to move along a straight trajectory [15,17]. Another such factor may be the tendency to minimize neural effort for interaction torque control by producing movements through active rotation of a single joint and allowing the other joint to be driven by interaction torque [18,19]. The load attached at the distal portion of the arm required larger muscle effort for movement production, and thus, it increased the weight of muscle effort in the resultant cost function, making the contribution of this factor to movement formation more apparent.

### Cost function representation of muscle effort

The interpretation that the increased frequency of transverse strokes caused by load represented minimization of muscle effort is consistent with the finding that the strokes in the preferred directions were optimized in terms of *I*_*INTS*_, *I*_*IR*_, and *I*_*E*_, i.e. the cost functions each of which may represent muscle effort in some way. However, only the *I*_*INTS*_ optimization was enhanced by the load, as was revealed by the analyses of mean values of the cost functions and of the percentage of strokes optimized by each cost function. No apparent effect of the load on optimization of *I*_*IR*_ was found. The following considerations show that the preference to minimize muscle effort may be better represented by *I*_*INTS*_ than by *I*_*IR*_. The directions optimal in terms of *I*_*IR*_ are perpendicular to the forearm [25], and therefore, they are achieved through single-joint elbow movements. These movements require muscle effort for compensation for interaction torque to fixate the shoulder. The directions optimal in terms of *I*_*INTS*_ are also achieved through active elbow motion but they require zero muscle effort for interaction torque control at the shoulder. The directions optimal in terms of *I*_*INTS*_ have low inertial resistance because they are close to those optimal in terms of *I*_*IR*_. Low inertial resistance and no muscle effort for shoulder control may make the directions in which *I*_*INTS*_ is optimized more economical than the directions in which *I*_*IR*_ is optimized. Thus, movements optimized in terms of *I*_*INTS*_ may have multiple benefits, including low muscle effort and low neural effort for interaction torque control at the shoulder.

No load effect was found for *I*_*E*_ either. In contrast to *I*_*INTS*_ and *I*_*IR*_, optimization of this cost function depended on the arm. The number of strokes optimized in terms of *I*_*E*_ was higher in the dominant arm compared with the nondominant arm. Also, inter-subject variability of this characteristic was high (Table 2). These results suggest that in addition to muscle effort, this cost function may depend on other factors. Indeed, this cost function represents mechanical energy of motion [14]. It is possible that the same muscle effort can produce different mechanical energy, depending on organization of control. For example, efficient exploitation of interaction torque may allow the dominant arm to generate larger mechanical energy through the same muscle effort compared with the nondominant arm. This interpretation accounts for the results obtained for *I*_*E*_, suggesting that mechanical energy is minimal in the directions in which muscle effort is minimal and it is lower in the nondominant arm due to inefficient movement control compared with the dominant arm.

The discussed results highlight *I*_*INTS*_ as the cost function that represents muscle effort best out of the considered cost functions. However, it should be taken into account that all cost functions used in this study to assess muscle effort were computed through muscle torque that may be not an adequate characteristic of muscle effort. For example, muscle torque can be low when antagonistic muscles are activated to a high level. Also, cost functions relying on muscle torque do not take into account many factors that influence muscle energy expenditure, such as muscle contractile dynamics, recruitment of slow vs. fast motor units, elastic properties of tissues surrounding each joint, etc. Taking into account the complexity of factors influencing muscle energy expenditure, any formal cost function would have certain limitations. For this reason, results obtained with the use of cost functions need to be verified through experimental manipulations that directly target the studied phenomenon, like applying the distal load in the present study. Another example is using hypergravity to support muscle torque impulse minimization during upward and downward arm movements [9]. In particular, experimental verification needs to be performed for the weighted sum of *I*_*E*_ and *I*_*ACC*_ suggested in [14] as a cost function optimized during arm movements. The lack of support obtained in our study emphasizes the need for further validation of this cost function.

### Effect of arm dominance

The tendency to move in the transverse directions was similar in the dominant and nondominant arm. Indeed, the load increased the bias strength and the percentage of strokes in quadrants I and III equally in both arms. In addition, the mean values of *I*_*INTS*_ and the percentage of strokes optimized by *I*_*INTS*_ increased with load in both arms. The lack of interlimb differences in all these characteristics suggests that the tendency to minimize muscle effort was independent of the arm dominance.

The similarity with which the dominant and nondominant arm responded to the load was in contrast to interlimb differences in control of interaction torque. Interaction torque emerges at the limb’s joints due to mechanical interactions among limb segments [46]. A decreased ability of the nondominant arm to control interaction torque is well-recognized (for review, see [47]). Consistent with this knowledge, it was demonstrated that during the free-stroke drawing task, the portion of strokes optimized by *I*_*INTE*_ and *I*_*INTS*_ (i.e. strokes during which interaction torque was exploited for rotation of the elbow or shoulder, respectively) was substantially lower in the nondominant arm compared with the dominant arm [40]. Even though directional preferences of the nondominant arm revealed in that study were mirror-symmetric and optimization of *I*_*INTE*_ and *I*_*INTS*_ accounted for directional preferences in both arms, the nondominant arm failed to exploit interaction torque for production of subordinate joint motion in the preferred directions.

The results obtained for *I*_*INTE*_ in the present study were in agreement with the findings of [40]. Both the mean value of *I*_*INTE*_ and the portion of strokes optimized by this cost function were substantially lower in the nondominant arm compared with the dominant arm (Figure 6a and Table 2). This result confirmed that the nondominant arm did not exploit interactions torque caused by shoulder motion at the elbow as effectively as the dominant arm did. In contrast to the results obtained in [40], no significant arm effect was observed for *I*_*INTS*_ in the present study, either for its mean value or for the portion of strokes optimized by this cost function. The load caused similar increases in these characteristics in both arms. The difference between the two studies with respect to the results for *I*_*INTS*_ is consistent with the idea discussed in the previous section that this cost function may be optimized for more than a single reason. It may increase due to both minimization of muscle effort and minimization of neural effort for control of interaction torque at the shoulder [18,19,31,40].

The similar effect of load on the directional preferences suggests similarity in planning for the required muscle effort in the dominant and nondominant arm. The generation of muscle effort may also be similar in both arms, as suggested by approximately equal increases in the *I*_*INTS*_ characteristics caused by load in each arm. However, the nondominant arm may exploit the generated muscle effort during movement execution less effectively compared with the dominant arm. This interpretation is supported by the less proficient interaction torque control along the longitudinal direction as well as by the lower values of *I*_*E*_ in the nondominant arm compared with the dominant arm found in the present study. This interpretation is also consistent with the previously recognized deficiency of the nondominant arm in interaction torque control [38-40].

## Conclusions

To summarize, distal load increased the tendency to produce arm movements in the transverse directions. Since the load required higher muscle effort for movement production in all directions, the changes in directional preferences suggest that the load enhanced the tendency to minimize muscle effort. Consistent with this interpretation, three cost functions (*I*_*INTS*_, *I*_*IR*_ and *I*_*E*_) that may represent muscle effort were optimized in the transverse directions. The findings support the contribution of muscle effort minimization to movement planning. This contribution may be relatively small during movements without load and it increases with load. This conclusion is consistent with a theory that the resultant cost function optimized during arm movements is complex and it includes several components weights of which change depending on movement conditions. The increases in the preference to produce strokes in the transverse directions caused by load were similar in the dominant and nondominant arm. This finding is important because it reveals differences in control of muscle effort compared with control of interaction torque in which the nondominant arm is deficient.

## Competing interests

Both authors declare that they have no competing interests.

## Authors' contributions

WW carried out data analysis and was critically involved in the design and conduction of the experiment and, in the manuscript preparation. ND made critical contribution to development of conception and design of the study, data analysis, and manuscript preparation. Both authors read and approved the final manuscript.
